# Editorial: New challenges and future perspectives in motivation and reward

**DOI:** 10.3389/fnbeh.2023.1293938

**Published:** 2023-09-27

**Authors:** Wenqiang Chen, Hongbin Yang

**Affiliations:** ^1^Section of Integrative Physiology and Metabolism, Joslin Diabetes Center, Boston, MA, United States; ^2^Department of Medicine, Harvard Medical School, Boston, MA, United States; ^3^Department of Affiliated Mental Health Center of Hangzhou Seventh People's Hospital and School of Brain Science and Brain Medicine, Zhejiang University School of Medicine, Hangzhou, China; ^4^Liangzhu Laboratory, State Key Laboratory of Brain-machine Intelligence, MOE Frontier Science Center for Brain Science and Brain-Machine Integration, Zhejiang University, Hangzhou, China; ^5^NHC and CAMS Key Laboratory of Medical Neurobiology, Zhejiang University, Hangzhou, China

**Keywords:** reward, motivation, drug use, binge drinking, impulsivity

Motivation plays a crucial role in one's connection with the world. Internal desires (internal drive) or environmental stimuli (external drive) in our daily lives, such as the energetic refreshment and pleasurable sensations following sports, drive our actions toward becoming reinforced behaviors. Therefore, motivated behaviors, occurring in pursuit of goals, are essential for the survival of species including humans. This is because not only can basic physiological needs like food and water be obtained through motivated behaviors, but also their maladaptive manifestations, such as binge drinking and drug seeking, can impose a significant burden on families and society.

Behavior can be motivated by rewarding or aversive stimuli. In the realm of social and behavioral cognition, the interaction between motivation and reward or aversion influences learning, although there is debate regarding whether reward has a detrimental impact on motivation (Jovanovic and Matejevic, 2014). In the past, Pavlov's classical operant conditioning has provided a compelling framework for understanding how an individual's behavioral response to reward or aversion can produce learning (Pavlov, 2010; de Jong et al., 2019). However, decades later, the theory of “reward prediction errors” emerged, assigning a central role in reward processing to the midbrain dopamine system (Schultz, 2016). Consequently, the overarching theme in the field of motivation and reward revolves around the neural systems that integrate intrinsic and extrinsic signals to encode reward-related information.

In this Frontier Research Topic “*New Challenges and Future Perspectives in Motivation and Reward*,” the authors shed light on innovative research in the field of motivation and reward. Here, this editorial provides a summary of key findings from each published article. Firstly, Zhang et al. provide an overview of the rewarding effects of “*blind box*” products on consumers' emotions and cognition. Taking the perspective of reward uncertainty, the authors conducted questionnaires and analyses to explore how four variables—perceived uncertainty, perceived luck, curiosity, and impulsive purchase intention—could influence decision-making. The results indicate that among these variables, perceived uncertainty significantly predicts impulsive purchase intentions, and this effect is positively moderated by perceived luck. This suggests that during a “blind box” purchase, consumers' motivation can be significantly influenced by curiosity and perceived luck. Additionally, the authors recommend that enterprises learn from the theoretical model of “uncertain rewards” to stimulate purchase motivation when launching a new product line.

Although the uncertainty of “blind box” items can be rewarding and lead to impulsive purchases, financial constraints can usually cause stress. Under stress, individuals may tend to consume more alcohol and illicit drugs. The following two articles used rodent models and explored how stress and impulsivity can mechanistically influence motivated behaviors. McCarthy et al. compared the effects of exposure to two types of stressors, namely chronic stress and sub-chronic stress, on the increased motivation in binge-like alcohol consumption. The researchers discovered that only exposure to chronic, not sub-chronic stress, led to increased alcohol intake. In the brain, the mesolimbic pathway, especially the nucleus accumbens (NAc), is considered as central to reward processing (Lammel et al., 2014; Klawonn and Malenka, 2018; Yang et al., 2018). Furthermore, the authors illustrated the critical involvement of the NAc adenosine 2A receptor in the link between stress and binge drinking. This suggests that additional strategies aimed at activating this receptor could potentially reduce alcohol consumption and, consequently, alleviate the burden of stress-related disorders.

Excessive alcohol consumption can result in numerous health risks, however, the use of illicit drugs like cocaine can pose even more serious consequences, affecting not only individuals who use these drugs but also their families and communities. Arrondeau et al. utilized two strains of rat, roman high- (RHA) and low-avoidance (RLA) rats, which innately exhibit phenotypical differences in avoidance behavior and susceptibility to drug abuse. Employing a rat gambling task, the researchers measured the baseline levels of motor impulsivity and risk-related impulsive choice in these rats. Subsequently, these rats were exposed to cocaine self-administration, a paradigm considered to be a golden standard for modeling drug-seeking behaviors in rodents (Bhattacherjee et al., 2019). The results indicate that at baseline, motor impulsivity is positively associated with risk-related impulsive choice. Further, in RHA rats that exhibit high motor impulsivity, increased drug-seeking behaviors are observed, suggesting that motor impulsivity can predict the extent of drug abuse and drug-primed relapse. Of note, the researchers showed that the use of the dopamine stabilizer, aripiprazole, can effectively inhibit cocaine-primed drug-seeking behaviors in both RHA and RLA rats, acting on dopamine D_2/3_ receptors. Thus, this study demonstrates that motor impulsivity serves as a robust predictor of vulnerability to drug-seeking, underscoring the importance of investigating maladaptive motivated behaviors in the treatment of individuals with a heightened vulnerability to drug abuse.

While alcohol and drug use are rewarding, food consumption can also produce satisfactory feelings and thereby influencing motivation. Motivated feeding behaviors comprise two phases: the appetitive phase and the consummatory phase (Keen-Rhinehart et al., 2013). During the appetitive phase, animals actively seek and come into contact with food through foraging and hoarding, while in the consummatory phase, animals consume the foods by chewing and swallowing. Champeil-Potokar et al. dissected these phases of food consumption and explored whether production of ultrasonic vocalizations (USVs), which are closely linked to affective states and physiological needs in rodents during social interaction, can predict various phases of feeding behaviors. Previously, subtypes of USVs have been identified to reflect specific behavioral patterns. For instance, a 50-kHz USV is typically associated with activation of brain reward systems, while a 20-kHz USV is considered indicative of aversive behaviors. Using a comprehensive ethogram of behaviors, the authors recorded USVs produced during each syllable. They showed that rats emit 40-kHz flat USVs during feeding, particularly during chewing, suggesting the significance of taking the social context into account when studying motivated behaviors related to appetitive reward.

In conclusion, the articles in this Research Topic make a substantial contribution to enhancing our understanding of neural basis of motivation and reward. This marks an important stride toward gaining novel insights into mood and cognition.

## Author contributions

WC: Writing—original draft, Writing—review and editing. HY: Conceptualization, Supervision, Validation, Writing—review and editing.
